# When myelin breaks, tau aggregates — a new perspective on Alzheimer's disease

**DOI:** 10.1002/alz.71774

**Published:** 2026-08-19

**Authors:** Assia Tiane, Daniel van den Hove, Tim Vanmierlo

**Affiliations:** ^1^ Department of Psychiatry and Neuropsychology, Mental Health and Neuroscience Research Institute (MHeNs) Maastricht University Maastricht The Netherlands; ^2^ Department of Neuroscience, Biomedical Research Institute, Faculty of Medicine and Life Sciences Hasselt University Hasselt Belgium

**Keywords:** Alzheimer's disease, microglia, myelin, oligodendrocytes, tau pathology

## Abstract

Increasing evidence suggests that myelin dysfunction and oligodendrocyte pathology are active contributors to neurodegeneration. In Alzheimer's disease (AD), the link between tau aggregation and myelin integrity remains unclear, despite the preferential emergence of tau pathology in late‐myelinating regions. Here, we propose a myelin‐centered framework for tau pathology based on three mechanisms. First, vulnerability of late‐myelinating oligodendrocytes may drive myelin breakdown, metabolic stress, and axonal dysfunction, promoting tau hyperphosphorylation. Second, microglial responses to myelin injury may become maladaptive, with lipid overload impairing tau clearance. Third, oligodendrocytes may act as conditional reservoirs facilitating tau propagation across myelinated networks. Together, these processes suggest that myelin loss may contribute to tau accumulation, clearance deficits, and spread, providing a framework for future experimental testing. This perspective highlights myelin biology as a source of new conceptual insights and therapeutic strategies in AD.

## Highlights

- Myelin changes may contribute to tau pathology in Alzheimer's disease (AD)
- Oligodendrocyte vulnerability links myelin breakdown to tau changes
- Myelin injury induces microglial states that impair tau clearance
- Oligodendrocytes may act as reservoirs for tau propagation
- Myelin preservation emerges as a therapeutic strategy in AD

## 1 RETHINKING TAU PATHOLOGY THROUGH MYELIN VULNERABILITY

Once considered a passive electrical insulator, myelin is now recognized as a dynamic regulator of neuronal signaling (adaptive myelination), metabolic support, and tissue repair, placing it at the forefront of modern neuroscience. Increasing evidence indicates that myelin dysfunction and oligodendrocyte pathology are not merely secondary consequences but potential active contributors in a wide spectrum of neurodegenerative diseases, including Alzheimer's disease (AD).^1^ This paradigm shift reframes myelin as a key participant in both neurodegeneration and repair and, as such, as a promising target for the development of novel treatment strategies for neurodegenerative disorders such as AD.

A central pathological hallmark of many neurodegenerative disorders is the accumulation of misfolded proteins, such as amyloid beta (Aβ) plaques and tau neurofibrillary tangles.^2^ Although these aggregates are well‐established drivers of neuronal loss, their impact on, and relationship with, myelin structure and dynamics remain relatively understudied. In the case of Aβ, recent work has demonstrated that myelin loss can actively promote Aβ deposition in experimental mouse models.^3^ The relationship between tau aggregation and myelin integrity is far less understood, leaving a critical gap in our understanding of how tauopathies influence myelin pathology, or vice versa.

Developmental myelination of the central nervous system (CNS) begins prenatally and continues into early adulthood. This process follows a highly conserved spatiotemporal pattern, proceeding in a caudal‐to‐rostral and sensory‐to‐motor manner: myelination starts in the spinal cord and brainstem, extends through the cerebellum, and progresses along subcortical pathways. The last regions to mature are higher‐order association cortices, such as the prefrontal cortex, which continue to myelinate throughout adolescence and into early adulthood.^4^ This spatiotemporal pattern reflects functional necessity, as systems essential for survival myelinate first, while higher‐order cognitive regions myelinate last.

**FIGURE 1:**
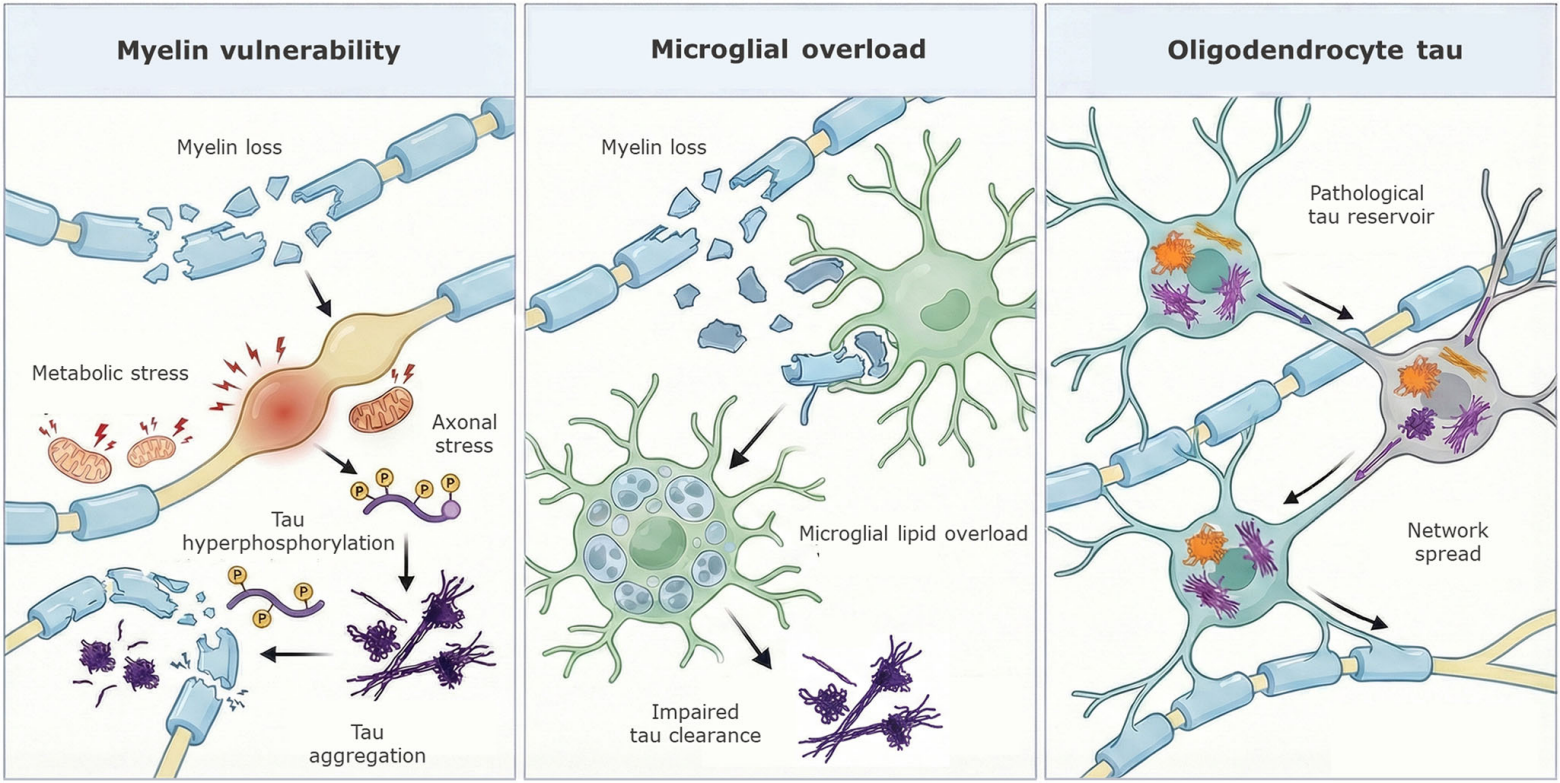
Myelin‐centered mechanisms linking myelin damage to tau pathology in Alzheimer's disease (AD). Schematic illustration of three pathways by which myelin dysfunction may promote tau pathology. Left: Intrinsic vulnerability of myelin, particularly in late‐myelinating regions, leads to myelin loss, metabolic stress, and axonal dysfunction, creating conditions that favour tau hyperphosphorylation and aggregation. Middle: Myelin damage generates excessive lipid‐rich debris that diverts microglial resources toward myelin clearance, potentially driving lipid overload and reducing microglial capacity to remove pathological tau. Right: Under conditions of myelin stress, oligodendrocytes may accumulate pathological tau and act as conditional reservoirs that facilitate tau propagation along myelinated networks. The pathways depicted represent a conceptual framework based on current evidence and highlight several mechanisms that remain to be experimentally validated. Together, these mechanisms provide a conceptual framework through which myelin dysfunction could influence tau accumulation, clearance, and spread during AD progression.

Of interest, in as early as 1996, Braak and Braak noted that the pattern of tau spread in AD mirrors the inverse sequence of cortical myelination across development.^5^ This observation aligns with the retrogenesis model of AD, which proposes that myelin degeneration progresses in the reverse order of myelin formation.^6^ Together, these insights point to a potential mechanistic link between myelin vulnerability and tau aggregation and spreading in AD. Here, we propose three possible working models for this mechanistic link (Figure 1): (1) Myelin damage as a direct driver of tau pathology; (2) microglia as gatekeepers linking myelin damage to tau pathology; or (3) oligodendrocytes as reservoirs of tau pathology.

Of note, these models should not be interpreted as evidence that myelin dysfunction is the sole or primary driver of tau pathology. Alternative processes, including aging‐related cellular decline, vascular dysfunction, chronic neuroinflammation, mitochondrial impairment, and systemic metabolic disturbances, may independently contribute to both myelin injury and tau accumulation.^7^, ^8^, ^9^ Likewise, increasing evidence indicates that pathological tau itself can disrupt oligodendrocyte function and compromise myelin integrity.^10^ Thus, rather than proposing a strictly unidirectional relationship, we view myelin dysfunction and tau pathology as components of a potentially bidirectional pathogenic cycle that warrants further investigation.

## 2 MYELIN DAMAGE AS A DIRECT DRIVER OF TAU PATHOLOGY

If late‐myelinating regions are intrinsically more vulnerable to both myelin breakdown and tau accumulation, an important question arises: which process occurs first? Evidence suggests that myelin disruption may precede and potentially facilitate tau pathology. Couttas et al. showed that defects in myelin biosynthesis in cortical regions already occur in the preclinical stage of AD, before the emergence of neurofibrillary tangle pathology.^11^ If myelin degradation promotes tau pathology, then regions with high baseline myelination should, in theory, be relatively protected from tau aggregation. Supporting this idea, imaging work by Rubinski et al. demonstrates that greater myelin content is associated with increased resistance to tau pathology in AD.^12^ Specifically, the authors observed that cortical regions with higher myelination exhibit a lower tau‐PET (positron emission tomography) signal, suggesting a protective role for intact myelin. Conversely, regions characterized by high myelin turnover, such as those undergoing adaptive myelination, may be particularly vulnerable to myelin impairment and its downstream consequences for tau pathology.

Further support for a causal link between myelin disruption and tau pathology comes from demyelinating diseases outside the AD spectrum. Abnormal tau phosphorylation and accumulation of insoluble tau species have been observed in multiple sclerosis (MS).^13^ Notably, Anderson et al. demonstrated that both experimental models of MS and human demyelinated MS lesions exhibit pronounced tau hyperphosphorylation.^14^, ^15^ Although MS differs etiologically from AD, these findings demonstrate that demyelination per se is sufficient to trigger pathological tau modifications.

These observations raise the question of how myelin disruption mechanistically facilitates tau pathology at the molecular and cellular level. Even though strong evidence on how myelin damage may lead to tau hyperphosphorylation is lacking, previous findings makes it tempting to speculate myelin breakdown is a primary driver.

Bartzokis et al. proposed that late‐myelinating oligodendrocytes, responsible for the prolonged process of intracortical myelination, may represent a particularly vulnerable cellular population in the adult brain.^16^ Compared to early‐myelinating oligodendrocytes, they are thought to be less efficient at myelin production and tend to generate thinner myelin sheaths, which may be more susceptible to age‐ and disease‐related damage. In parellel to their structural vulnerability, late‐myelinating oligodendrocytes progressively accumulate iron and cholesterol, a feature that becomes more pronounced with aging.^16^ Elevated iron levels can promote oxidative stress and dysregulate kinase and phosphatase activity, thereby facilitating tau hyperphosphorylation and aggregation.^17^ Together, these properties suggest that regions enriched in late‐myelinating oligodendrocytes are predisposed to myelin breakdown and may provide a permissive environment for the initiation and propagation of tau pathology, while also rendering these regions intrinsically more vulnerable to further myelin damage in response to local tau aggregation and deposition.

Beyond their role in enabling saltatory conduction, oligodendrocytes provide essential metabolic support to neurons.^18^ They supply energy metabolites such as lactate and pyruvate, which serve as substrates for neuronal adenosine triphosphate (ATP) production. The transfer of these metabolites occurs through cytoplasmic channels within the myelin sheath and is mediated by monocarboxylate transporters that facilitate metabolic coupling between oligodendrocytes and axons.^19^, ^20^ Disruption of myelin integrity therefore compromises this oligodendrocyte‐derived metabolic support. Given that neurons are among the most energy‐demanding cells in the brain, disturbances in their metabolic homeostasis can have profound consequences. Accordingly, bioenergetic impairments have been identified as important triggers of tau pathology in neurodegenerative disease.^19^ Chronic axonal energy stress and mitochondrial dysfunction activate tau‐modifying kinases and impair tau clearance pathways, thereby favoring tau hyperphosphorylation and aggregation.^21^, ^22^ Overall, defective myelin bioenergetics disrupts metabolic coupling within the axon–myelin unit. As this interaction is critical for maintaining axonal energy homeostasis, demyelination‐associated dysfunction reduces mitochondrial efficiency, promotes tau pathology, and ultimately contributes to axonal injury.

Loss of myelin not only deprives axons of metabolic support but also profoundly perturbs the axonal cytoskeleton.^23^ Myelin disruption and the resulting axonal stress have been associated with reduced expression of cytoskeletal and transport proteins such as kinesin light chain and neurofilaments, as well as alterations in axonal transport dynamics. This suggests that demyelination itself undermines cytoskeletal integrity.^24^ In AD and related tauopathies, aberrant phosphorylation of tau reduces its affinity for microtubules, leading to tau detachment and microtubule destabilization, which in turn impairs kinesin‐ and dynein‐mediated transport and promotes further cytoskeletal breakdown.^25^, ^26^ The resulting structural instability and transport deficits create a permissive environment for pathological tau accumulation. This is reflected in demyelination‐associated models where axonal damage coincides with tau hyperphosphorylation and activation of tau kinases such as cyclin‐dependent kinase 5 (CDK5).^27^ These studies collectively support a model in which myelin loss destabilizes the axonal cytoskeleton, thereby facilitating pathological modifications of tau and amplifying neurodegenerative cascades.

## 3 MICROGLIA AS GATEKEEPERS LINKING MYELIN DAMAGE TO TAU PATHOLOGY

In recent years, there has been a substantial shift toward recognizing microglia as central regulators of AD pathophysiology.^28^ The identification of disease‐associated microglia (DAM) has provided critical insight into how microglial states dynamically evolve during disease progression.^29^ In the presence of pathological stimuli, such as Aβ plaques and misfolded tau aggregates, microglia become activated and initiate protective responses aimed at clearance. However, as pathology accumulates and the disease progresses, sustained microglial activation can transition from an initially beneficial state to a maladaptive one, characterized by chronic inflammation, impaired clearance capacity, axonal stress, and progressive neurodegeneration.^9^

Activated microglia are classically viewed as mediators of Aβ plaque and tau aggregate clearance. However, in principle, these activated cells could also be redirected toward myelin as a phagocytic substrate. Such misdirected microglial activity has been proposed as a potential contributor to myelin damage in AD.^30^, ^31^ To date, however, direct evidence supporting tau‐driven myelin phagocytosis in AD remains limited. Moreover, multiple studies indicate that myelin abnormalities are already present at very early stages of AD, preceding the emergence of overt tau pathology.^32^, ^33^, ^34^ These observations argue against a model in which microglia‐driven myelin loss is merely a downstream consequence of tau aggregation and instead support the notion that myelin damage may represent an early and upstream event in the disease process.

Rather than acting as primary drivers of myelin loss, microglia may be more accurately positioned as responders to early myelin injury, with important downstream consequences for tau pathology and disease progression. In this context, a distinct microglial subpopulation termed lipid‐droplet‐accumulating microglia (LDAM) has been identified in the aging brain by Marschallinger et al.^35^ LDAMs are present in hippocampal tissue from patients with AD and are characterized by excessive lipid droplet accumulation, elevated reactive oxygen species (ROS) production, and impaired phagocytic capacity.^36^ These features suggest that lipid overload fundamentally alters microglial function.

Myelin debris represents a particularly lipid‐rich substrate, and excessive myelin degradation may therefore divert microglial resources away from the efficient clearance of pathological proteins such as tau. Although a direct link between myelin debris accumulation and defective tau clearance has not yet been conclusively demonstrated, studies such as Depp et al. suggest that myelin pathology can substantially redirect microglial activity and priorities.^3^ This provides a plausible mechanistic framework through which chronic myelin damage could indirectly influence the handling of pathological tau species. When myelin degradation exceeds the capacity of microglial lysosomal processing, excessive lipid and cholesterol uptake induce lysosomal stress, metabolic dysfunction, and lipid droplet accumulation within microglia.^37^ This overloaded state has been associated with reduced degradative efficiency and impaired phagocytosis of alternative substrates, including Aβ and aggregated tau. Consistent with this mechanism, tauopathy models show that lipid‐laden (LDAM) microglia exhibit heightened oxidative stress, a pro‐inflammatory phenotype, and diminished tau clearance, thereby exacerbating neurodegeneration.^38^ Together, these findings support a model in which early myelin injury drives microglial lipid overload, locking microglia into a dysfunctional state, thereby indirectly facilitating tau accumulation and spread by overwhelming microglial clearance pathways.

## 4 OLIGODENDROCYTES AS RESERVOIRS OF TAU PATHOLOGY

Although expressed at lower levels than in neurons, tau is also endogenously expressed in mature oligodendrocytes, whereas it is largely absent from microglia and astrocytes.^39^ In oligodendrocytes, tau contributes to microtubule organization and process extension during differentiation and myelin formation, indicating a functional, cell‐intrinsic role. Disruption of tau homeostasis, through hyperphosphorylation or mislocalization, impairs oligodendrocyte function and myelin integrity.^40^ Yet, when myelin is under stress—potentially driven by oxidative stress, lipid dysregulation, and metabolic imbalance—disruption of oligodendrocyte‐intrinsic tau homeostasis may not only impair myelin integrity locally, but also create conditions that favor abnormal tau handling and release, potentially contributing to wider tau propagation.^41^ Recent evidence further suggests that oligodendrocytes can produce Aβ, albeit at lower levels than neurons.^42^ This raises the intriguing possibility that oligodendrocytes may participate in local amyloid biology in addition to tau homeostasis. Given the well‐established ability of Aβ and neuroinflammatory signaling to potentiate tau pathology, it will be important to determine whether oligodendrocyte‐derived Aβ contributes to cellular stress, altered axon–glia communication, or abnormal tau handling within oligodendrocytes themselves.^43^ Such interactions remain largely unexplored in AD.

Under conditions of myelin damage, oligodendrocytes experience profound cytoskeletal and metabolic stress as well as altered axon–glia signaling.^23^ Such stressors may disrupt tau phosphorylation dynamics and intracellular trafficking, potentially increasing tau retention or release from oligodendrocytes. Several transgenic animal models expressing mutant human tau demonstrate tau accumulation and aggregation within oligodendrocytes, accompanied by oligodendroglial dysfunction, myelin loss, and impaired axonal transport.^44^, ^45^ However, the extent of oligodendroglial involvement may depend on the cellular expression pattern and promoter driving tau expression. Although overt tau aggregates are rarely detected in oligodendrocytes in post‐mortem AD tissue compared with primary tauopathies, this absence may reflect differences in tau aggregation kinetics rather than a lack of involvement in disease pathology.^46^ Oligodendrocytes may more effectively process or export misfolded tau, potentially promoting its propagation across neural networks. Consistent with a potential role in tau propagation, inoculation studies have demonstrated that oligodendrocytes can participate in the cellular response to exogenous tau seeds and may contribute to the amplification of seeded tau pathology.^47^ Given the intimate association between myelinating oligodendrocytes and long‐projecting axons, even subtle alterations in oligodendroglial tau handling could influence the spread of pathological tau species across neural networks.^48^ Furthermore, it is noteworthy that bridging Integrator 1 (BIN1), one of the genetic risk factor for sporadic AD and a well‐established modulator of tau pathology, is predominantly expressed in mature oligodendrocytes.^46^

Together, these observations raise the possibility that oligodendrocytes act as secondary reservoirs or modulators of pathological tau under specific disease conditions. Although oligodendroglial tau aggregation is a defining feature of several primary tauopathies, whether a comparable, but less pronounced, oligodendrocyte contribution exists in AD—potentially triggered by myelin stress rather than tau mutations− remains an important open question.

## 5 CONCLUDING REMARKS

Accumulating evidence challenges the long‐standing neuron‐centric view of tau pathology in AD and instead places myelin integrity at a critical intersection between neurodegeneration and disease progression. Here, we propose that myelin dysfunction may represent an underappreciated contributor to tau pathology rather than merely a downstream consequence of neurodegeneration. The preferential involvement of late‐myelinating regions, early myelin abnormalities preceding classical pathological hallmarks, and the convergence of microglial and oligodendrocyte responses to myelin injury collectively make it tempting to speculate that myelin breakdown may influence regional vulnerability to tau pathology and deserves further experimental investigation.

From this perspective, myelin loss may influence tau pathology through multiple, potentially synergistic mechanisms: by directly destabilizing the axon–myelin unit and cytoskeleton, by diverting microglial capacity toward excessive myelin debris processing and away from tau clearance, and by inducing stress responses in oligodendrocytes that alter tau handling and axonal support. Of note, these pathways do not need to operate independently but may converge to create a permissive environment for tau aggregation and spread, particularly within vulnerable cortical networks.

Ultimately, reframing AD through the lens of myelin biology opens new conceptual and therapeutic avenues. Interventions aimed at preserving myelin integrity, restoring axon–glia metabolic coupling, or preventing maladaptive glial responses to myelin injury, may represent promising strategies to slow or modify tau‐driven neurodegeneration. Recognizing when and why myelin breaks may therefore be key to understanding why and where tau aggregates and, ultimately, how to arrest the descent.

## CONFLICT OF INTEREST STATEMENT

The authors declare no conflicts of interest. Author disclosures are available in the Supporting Information.

## Supporting information




Supporting Information
